# Dissecting the Transcriptional Response to Elicitors in *Vitis vinifera* Cells

**DOI:** 10.1371/journal.pone.0109777

**Published:** 2014-10-14

**Authors:** Lorena Almagro, Pablo Carbonell-Bejerano, Sarai Belchí-Navarro, Roque Bru, José M. Martínez-Zapater, Diego Lijavetzky, María A. Pedreño

**Affiliations:** 1 Department of Plant Biology, Faculty of Biology, University of Murcia, Murcia, Spain; 2 Instituto de Ciencias de la Vid y del Vino (CSIC-Universidad de La Rioja-Gobierno de La Rioja), Complejo Científico Tecnológico, Logroño, Spain; 3 Department of Agrochemistry and Biochemistry, Faculty of Sciences, University of Alicante, Alicante, Spain; 4 Instituto de Biología Agrícola de Mendoza (CONICET-Universidad Nacional de Cuyo), Facultad de Ciencias Agrarias, Mendoza, Argentina; Huazhong University of Science & Technology(HUST), China

## Abstract

The high effectiveness of cyclic oligosaccharides like cyclodextrins in the production of *trans*-resveratrol in *Vitis vinifera* cell cultures is enhanced in the presence of methyl jasmonate. In order to dissect the basis of the interactions among the elicitation responses triggered by these two compounds, a transcriptional analysis of grapevine cell cultures treated with cyclodextrins and methyl jasmonate separately or in combination was carried out. The results showed that the activation of genes encoding enzymes from phenylpropanoid and stilbene biosynthesis induced by cyclodextrins alone was partially enhanced in the presence of methyl jasmonate, which correlated with their effects on *trans*-resveratrol production. In addition, protein translation and cell cycle regulation were more highly repressed in cells treated with cyclodextrins than in those treated with methyl jasmonate, and this response was enhanced in the combined treatment. Ethylene signalling was activated by all treatments, while jasmonate signalling and salicylic acid conjugation were activated only in the presence of methyl jasmonate and cyclodextrins, respectively. Moreover, the combined treatment resulted in a crosstalk between the signalling cascades activated by cyclodextrins and methyl jasmonate, which, in turn, provoked the activation of additional regulatory pathways involving the up-regulation of *MYB15*, *NAC* and *WRKY* transcription factors, protein kinases and calcium signal transducers. All these results suggest that both elicitors cause an activation of the secondary metabolism in detriment of basic cell processes like the primary metabolism or cell division. Crosstalk between cyclodextrins and methyl jasmonate-induced signalling provokes an intensification of these responses resulting in a greater *trans*-resveratrol production.

## Introduction

*Vitis vinifera* produces stilbenes, which are a small group of compounds characterized by a 1,2-diphenylethylene backbone. Most plant stilbenes are derivatives of the monomeric unit *trans*-resveratrol (*trans*-R; 3,5,4′-trihydroxystilbene). The formation of stilbenes is considered to be a part of the general defense mechanism since they display strong antifungal and antimicrobial activities [1]–[3]. In fact, *trans*-R is produced in both grapevine vegetative tissues and berries as well as in cell cultures in response to abiotic and biotic stress [1], [4]–[5]. Moreover, hundreds of studies have reported the beneficial effects of *trans*-R on neurological system [6], cardiovascular diseases [7], preventing carcinogenesis [8]–[9] and as an antiaging agent in the treatment of age-related human diseases [10].

Biosynthesis of *trans*-R starts from phenylalanine which is a key intermediate linking the primary metabolism and the secondary metabolism. Thus, the first step in the stilbene biosynthesis pathway consists in the transformation of phenylalanine into cinnamic acid in a reaction catalyzed by the enzyme phenylalanine ammonia lyase (PAL). The consecutive action of cinnamate 4-hydroxylase (C4H) and 4-coumarate-CoA ligase (4CL) transforms cinnamic acid into 4-coumaroyl-CoA, which is the common precursor of most of the phenolic compounds found in plants: lignins, flavonoids and stilbenoids. Then, one molecule of 4-coumaroyl-CoA is condensed with three malonyl-CoA units to produce either *trans*-R, through the action of stilbene synthase (STS), or naringenin chalcone by the action of chalcone synthase ([11] and references therein). Nowadays, different transcription factors (TFs) involved in the regulation of lignin, flavonol and anthocyanin metabolism are known [12]–[14]. The regulation of stilbene biosynthesis remains largely unknown, and only a role of TFs MYB14 and MYB15 has been recently reported in grapevine [15].

Stilbene biosynthesis is induced in response to a wide range of biotic and abiotic elicitors, which in turn, activate the expression of genes encoding stilbene biosynthesis pathway enzymes [16]–[17]. The activation of stilbene biosynthesis by particular elicitors is well-documented in grapevine, where the expression of *STS* genes and the production of stilbenes are induced upon elicitation with different fungal pathogens [18]–[19]. Stilbene biosynthesis is also triggered by signalling molecules such as methyl jasmonate (MJ) [20]. In this way, upon perception of jasmonate signals, the plant cell activates several defense mechanisms, reflected in a massive reprogramming of gene expression which leads to both the activation of stilbene biosynthesis and the expression of pathogenesis related-proteins (PR-proteins) [17], [21]. Moreover, the addition of MJ induces both the repression of cell cycle progression and the induction of phenylpropanoid metabolism in *Arabidopsis thaliana* cell cultures [22].

On the other hand, cyclodextrins (CD) are cyclic oligosaccharides that chemically resemble to the pectic oligosaccharides naturally released from the cell walls during a fungal attack [2]. They act as true elicitors since they provoke stilbene accumulation and induce the accumulation of new gene products like peroxidases, β-1,3-glucanases and chitinases [23]–[24]. Martínez-Esteso et al. [25] observed that enzymes from the *trans*-R biosynthesis pathway like STS were up-regulated by CD in grapevine (*V. vinifera* cv Gamay) cell cultures. In addition, CD are not only inducers of *trans*-R biosynthesis but also are promoters of adducts that remove *trans*-R from the culture medium, reducing the feedback inhibition and *trans*-R degradation, and allowing its accumulation at high concentrations [26]. Interestingly, Lijavetzky et al. [27] demonstrated that the combined use of CD and MJ enhanced the production of *trans*-R, which was strongly associated to an increased expression of *STS*, *PAL*, *C4H*, *4CL* genes in grapevine (*V. vinifera* cv Monastrell) cell cultures.

Global transcriptomic approaches can provide new clues on both the transcriptional cascade activated by elicitors and the possible interactions taking place when they are applied in combination. Considering the synergistic interaction of CD and MJ on *trans*-R production, we have transcriptionally analyzed the responses of grapevine cell cultures to the treatments with CD and MJ separately and in combination using the GrapeGen GeneChip. In this work, we identified regulators which are specifically activated in each treatment and discussed their possible involvement in the control of different physiological processes as well as the putative interaction of physiological and cellular responses giving rise to the synergistic accumulation of *trans*-R in the combined treatment.

## Results

### Cyclodextrins and methyl jasmonate synergistically induce *trans*-resveratrol production

**Figure 1:**
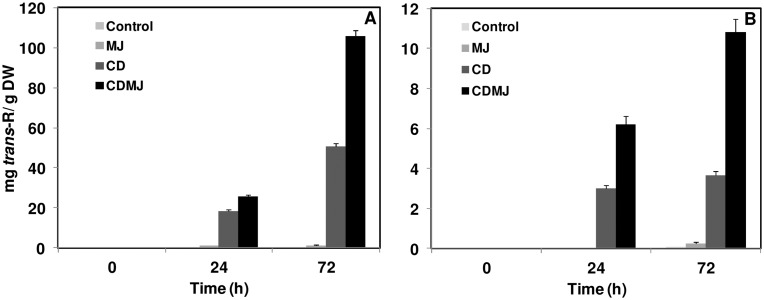
Effect of elicitation time course on *trans*-R (*trans*-resveratrol) production in grapevine cells treated with CD (cyclodextrins) and/or MJ (methyl jasmonate). (A) *trans*-R content in the extracellular medium. (B) *trans*-R content in the cells. Experiments were repeated three times. Data are the mean ± SD of the replicates.

In order to compare the effects of the different treatments on the production of *trans*-R in the cell cultures in which the transcriptomic analyses were carried out, the level of this compound was analyzed both in cells and extracellular medium at 0, 24 and 72 h. As shown in Figure 1A, a negligible amount of *trans*-R was detected in the extracellular medium when cell cultures were elicited with MJ (0.019 mg g^−1^ dry weight (DW) that means 0.19 mg L^−1^) in comparison with CD-treated cells. In contrast, the level of extracellular *trans*-R increased linearly up to 72 h in CD-treated cells (50.4 mg DW that means 504 mg L^−1^) and, when cells were simultaneously elicited with CD and MJ, the accumulation of *trans*-R in the medium increased more than twice (105.9 mg g^−1^ DW that means 1059 mg L^−1^) (Figure 1A). Similarly, the highest *trans*-R levels detected in the cells were reached in the presence of CD and MJ after 72 h of treatment (10.8 mg g^−1^ DW that means 108 mg L^−1^) (Figure 1B) although it was of one order of magnitude lower than the final concentration obtained in the medium.

### Global gene expression is differentially modulated by cyclodextrins and methyl jasmonate

**Figure 2:**
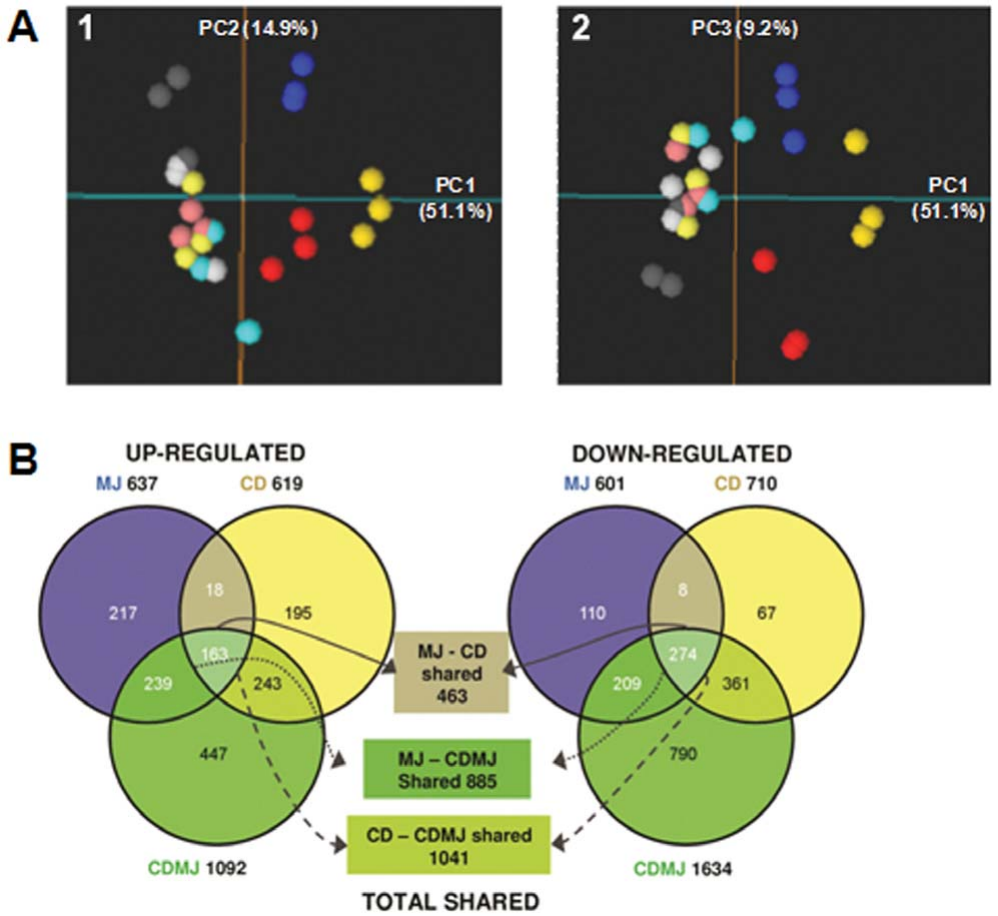
A, PCA over the expression dataset after the average of the redundant probesets expression values. **1,** PC1 vs PC2; **2,** PC1 vs PC3. Grey, Control; Blue, MJ (methyl jasmonate); Red, CD (cyclodextrins); Yellow, CD and MJ (cyclodextrins and methyl jasmonate); Light colors, just before cell treatments; Dark colors, 24 h cell treatments. **B,** **Genes significantly regulated by elicitor treatments.** C, control; MJ, methyl jasmonate; CD, cyclodextrins; CDMJ, cyclodextrins and methyl jasmonate. Venn diagram summary of significantly regulated transcripts. Non-redundant significant transcripts were obtained from probesets showing an at least 2-fold change and 5% FDR and *P*-value <0.05 for model variable in the corresponding control versus treatment 24 h series maSigPro comparison. Left side, up-regulated transcripts in each treatment; Right side, down-regulated transcripts in each treatment; Center, summary of significantly regulated transcripts shared by any two treatments.

RNA samples obtained from the four treatments (C, MJ, CD and CDMJ) at 0 and 24 h were used for the gene expression analyses. In order to validate the results obtained with the microarray analysis, a quantitative real time RT-PCR (qRT-PCR) assay on 7 differentially expressed transcripts was carried out using gene-specific primers (Table S1) based on the corresponding GrapeGen GeneChip probe set sequences. Linear regression analysis displayed highly significant correlations (average R^2^ = 0.96±0.02) for the 7 evaluated genes (Figure S1). Altogether, the experiment was considered highly reliable to support further transcriptomic analyses. Moreover, the results of a Principal Component Analysis (PCA) plot showed a strong consistency across biological replicas, and at 0 h, no apparent effect of elicitor addition was observed, since all the samples were grouped together (Figure 2A). Moreover, a consistent biological interpretation can be extracted from the three PCs, indicating that expression differences amongst samples were dominated by the elicitation response. The principal component 1 (PC1) explained 51.1% of the variation and displayed a similar trend in the response of the cells to all treatments (CD, MJ and CDMJ), although the combined treatment presented an intensified global expression response (Figure 2A). PC2 (14.9%) pointed to the specific effect of MJ, while PC3 (9.2%) showed the individual effect of CD and MJ which in turn, resulted to be opposite (Figures 2A.1 and 2A.2).

Expression changes over the whole dataset (Table S2) were analyzed by means of maSigPro time series comparisons with the control (C). In this way, we identified 3,659 differential probesets (5% FDR and two-fold change, Table S3), representing 3,306 non-redundant transcripts. Few differences were observed in the control between 0 and 24 h, while no significant differences were identified within the four samples at 0 h, even using a loose threshold (Bonferroni and Hochberg adjusted *P*-value <0.1; data not shown). These results demonstrate the homogeneity of the starting cell cultures for the four treatments, as it was previously shown by the PCA analysis (Figure 2A). Also consistent with the PCA results, most transcripts regulated in treatments with either CD or MJ were similarly regulated in the combined treatment, as it can be seen in the Venn diagrams comparing differentially expressed genes in each treatment (Figure 2B). In addition, a high proportion of significant transcripts were exclusively regulated by the joint action of CD and MJ.

With the aim of establishing the relationship amongst the particular responses to the treatments (MJ, CD, CDMJ), significant probesets were grouped according to their expression profiles in every treatment normalized to the control. According to a ‘gap’ analysis, 15 clusters were generated in a SOM analysis that allowed the identification of specific responses for each treatment as well as those shared by them (Figure S2; Table S4). According to the differentially expressed genes clustering, the most abundant expression profiles corresponded to the combined treatment-specific response (clusters 1 and 10), followed by responses shared by all three treatments (clusters 6 and 15) (Figure S2). Self-organizing maps (SOM) clustering analysis also identified a large proportion of transcripts co-regulated by one of the individual treatments as well as by CD and MJ, the proportion corresponding to CD separately or in combination with MJ (clusters 2, 9 and 14, Figure S2) was greater than that of MJ alone and in combination with CD (clusters 5 and 11, Figure S2). As it is shown in the PCA plot, the SOM analysis also revealed that the MJ-specific response (clusters 4 and 12) was stronger than that of CD (clusters 3 and 13) (Figure S2).

### Functional analysis of elicitor transcriptional responses

**Figure 3:**
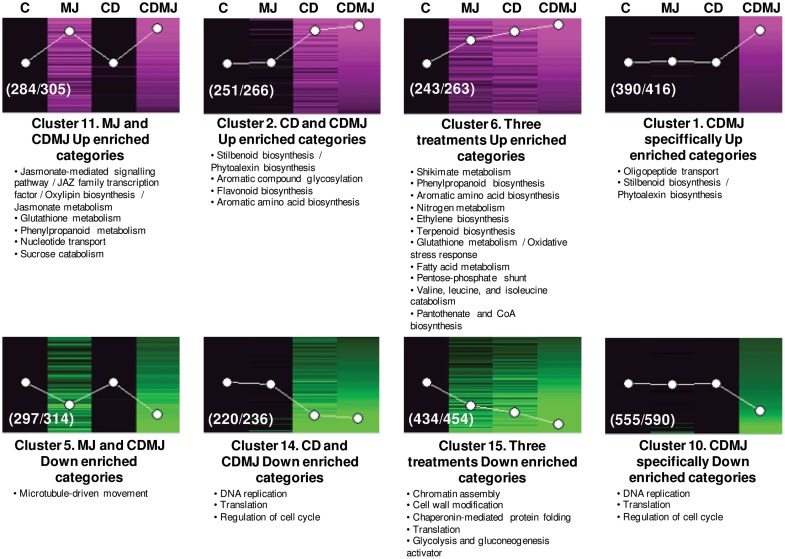
Functional categories enriched in clusters of transcripts sharing the same expression response to the treatments. Significant enrichment was only found in clusters 1, 2, 5, 6, 10, 11, 12, 14, and 15 from Figure S2. All non-redundant over-represented categories are listed below the corresponding cluster in order of significance except for related categories that are grouped in the same line. Significant enrichment according to Bonferroni-Hochberg adjusted *P*-value <0.05 in a Fisher’s exact test. Magenta and green intensity, level of up-regulated or down-regulated expression respectively, 24 h after treatment and normalized to the control; Black, transcripts not significantly regulated by the treatment. C, control; MJ, methyl jasmonate; CD, cyclodextrins; CDMJ; cyclodextrins and methyl jasmonate. The numbers in brackets indicate non-redundant transcripts/total probesets included in the cluster.

To understand the putative biological meaning of the different expression profiles detected, a FatiGO functional enrichment analysis (Figure 3) was performed in the transcripts grouped in each of the 15 SOM clusters (Figure S2). Significant functional enrichment (Bonferroni-Hochberg adjusted *P*-value <0.05 in a Fisher’s exact test) was only detected in the largest clusters (1, 2, 5, 6, 10, 11, 14 and 15; Figure 3) that corresponded to the transcript groups regulated by all treatments (clusters 6 and 15), only by CDMJ (1 and 10), by CDMJ and MJ (5 and 11) or by CDMJ and CD (2 and 14). Considering the functional categories enriched in cluster 6, the three treatments shared the activation of several pathways related with stilbene biosynthesis, including both the initial steps of the pathway and those of their precursors at different levels (i.e., nitrogen metabolism, shikimate biosynthesis, aromatic amino acid and phenylpropanoid biosynthesis; Figure 3 and Table S4). Other enriched categories in cluster 6 were ethylene and terpenoid biosynthesis, glutathione metabolism and the oxidative stress response, fatty acid metabolism, pentose-phosphate shunt, branched-chain amino acid catabolism, and pantothenate and CoA biosynthesis. In view of the expression trend in cluster 6, the magnitude of the activation of the mentioned processes was greater for CD than for MJ, and even greater for the combined treatment (Table S3).

On the other hand, jasmonate metabolism and signalling mediated by jasmonate were the most significantly enriched categories in the cluster of transcripts up-regulated by MJ as well as by the combined treatment (cluster 11, Figure 3). Within the same profile, a significant enrichment of other categories like the glutathione and phenylpropanoid metabolism, nucleotide transport and sucrose catabolism was found. As regards transcripts up-regulated only by CD and CDMJ (cluster 2, Figure 3), the functional enrichment analysis pointed to the activation of stilbenoid and flavonoid biosynthesis by CD as well as aromatic compound glycosylation and aromatic amino acid biosynthesis. The concurrent presence of CD and MJ synergistically activated stilbenoid biosynthesis, since this category was also over-represented in the CDMJ specifically up-regulated transcripts (cluster 1, Figure 3). Moreover, the expression of oligopeptide transporters was also specifically activated by the combined treatment.

The enriched functions within the transcripts repressed by the three treatments included basic cellular processes like chromatin assembly, translation, chaperonin-mediated protein folding and cell wall modification (cluster 15, Figure 3). As regards the genes repressed by MJ and CDMJ, an over-representation of the cytoskeleton organization (cluster 5, Figure 3) was observed. Finally, CD and CDMJ repressed DNA replication and the regulation of cell cycle-related processes, while some transcripts involved in those processes were repressed only by the combined treatment (cluster 14 and 10, respectively, Figure 3).

**Figure 4:**
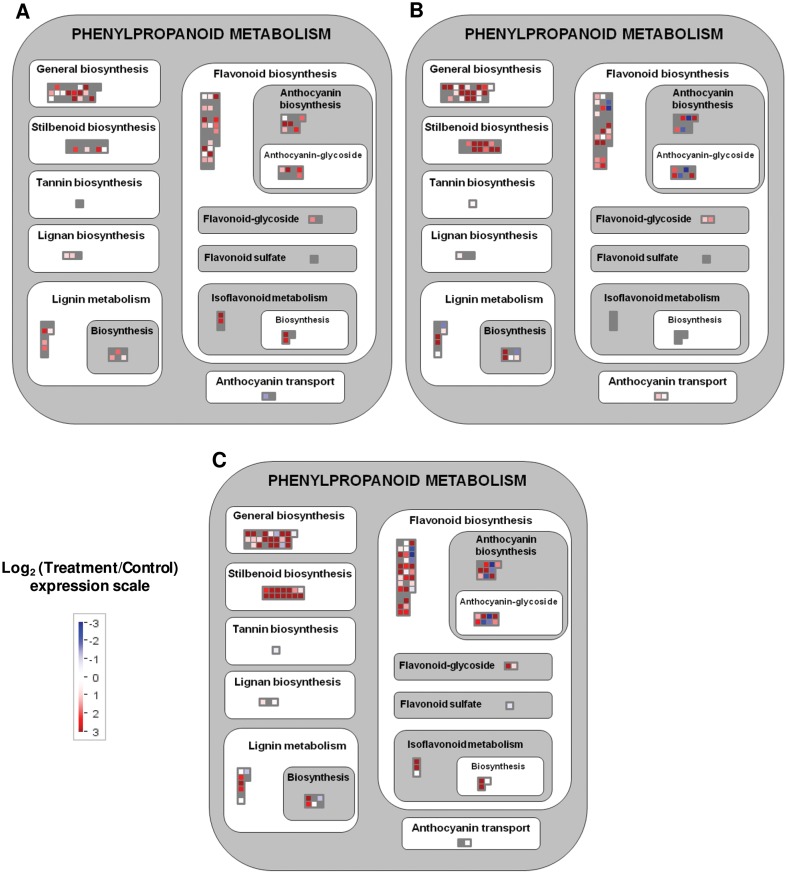
Mapman visualization of the significant genes in the ‘Phenylpropanoid metabolism’ pathway. A, MJ (methyl jasmonate); B, CD (cyclodextrins); C, CDMJ (cyclodextrins and methyl jasmonate). Resultant transcripts were considered after the average of significant redundant probesets expression values. Significant probesets according to a 5% FDR and *P*-value <0.05 for model variable in the corresponding control versus treatment 24 h series maSigPro comparison. Expression changes in the treatment normalized to these in the control are shown. Red, treatment up-regulated transcripts; blue, treatment down-regulated transcripts; grey, transcripts not significant in the treatment.

**Figure 5:**
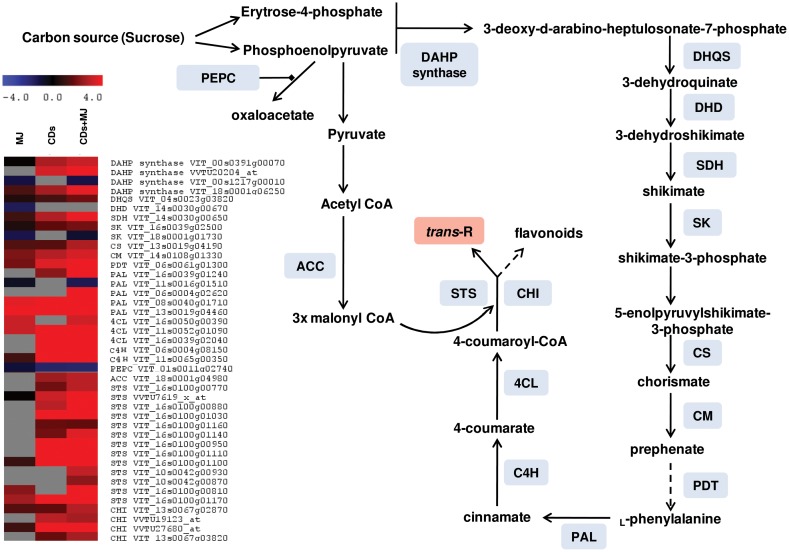
Modulation of *trans*-resveratrol biosynthesis pathway encoding enzymes gene expression by CD (cyclodextrins) and/or MJ (methyl jasmonate). Enzyme abbreviations: 4CL, 4-coumarate CoA ligase; ACC, acetyl-CoA carboxylase; C4H, cinnamate-4-hydroxylase; CHI, chalcone isomerase; CM, chorismate mutase; CS, chorismate synthase; DAHP synthase, 3-deoxy-D-arabino-heptulosonate 7-phosphate synthase; DHD, dehydroquinate dehydratase; DHQS, 3-dehydroquinate synthase; PAL, phenylalanine ammonia lyase; PDT, prephenate dehydratase; PEPC, phosphoenolpyruvate carboxylase; SDH, shikimate dehydrogenase; SK, shikimate kinase; STS, stilbene synthase.

A graphical overview of the main pathways altered by the different treatments was depicted using the MapMan software. Several components of the phenylpropanoid pathway were widely activated by the presence of elicitors (Figures 4 and 5 and Table S3). More particularly, CD separately or in combination with MJ activated a greater number of *PAL*, *4CL*, *C4H* and *STS* genes than MJ alone (Figure 5). Moreover, the activation of some phenylpropanoid biosynthesis precursors, like shikimate and aromatic amino acids, was significantly higher in cells treated with CD (separately or in combination with MJ) than in MJ-treated cells (Figure S3). In fact, CD and CDMJ strongly increased the expression of genes like 3-deoxy-D-arabino-heptulosonate 7-phosphate synthase (*DAHP*), 3-dehydroquinate synthase (*DHQS*), dehydroquinate dehydratase (*DHD*), shikimate dehydrogenase (*SDH*), shikimate kinase (*SK*), chorismate synthase (*CS*), chorismate mutase (*CM*), and prephenate dehydratase (*PDT*) (Figure 5). Interestingly, most of these genes were more highly induced by CDMJ than by CD. In contrast, MJ alone only provoked a slight increase in the expression of *DAHP, SDH, CS*, *CM* and *PDT* genes (Figure 5). Moreover, it is important to highlight that CD and MJ provoked the down-regulation of one gene encoding one phosphoenolpyruvate carboxylase (PEPC), whereas one acetyl-CoA carboxylase (ACC) involved in malonyl-CoA biosynthesis was only up-regulated in the presence of CD (Figure 5).

In relation to transport overview, the joint action of CD and MJ enhanced the expression of transcripts related with the transport of amino acids, oligopeptides and nucleotides (Figure S4). The activation of multidrug transporters was considerably higher in cells treated with MJ separately or in combination with CD than in CD-treated cells.

**Figure 6:**
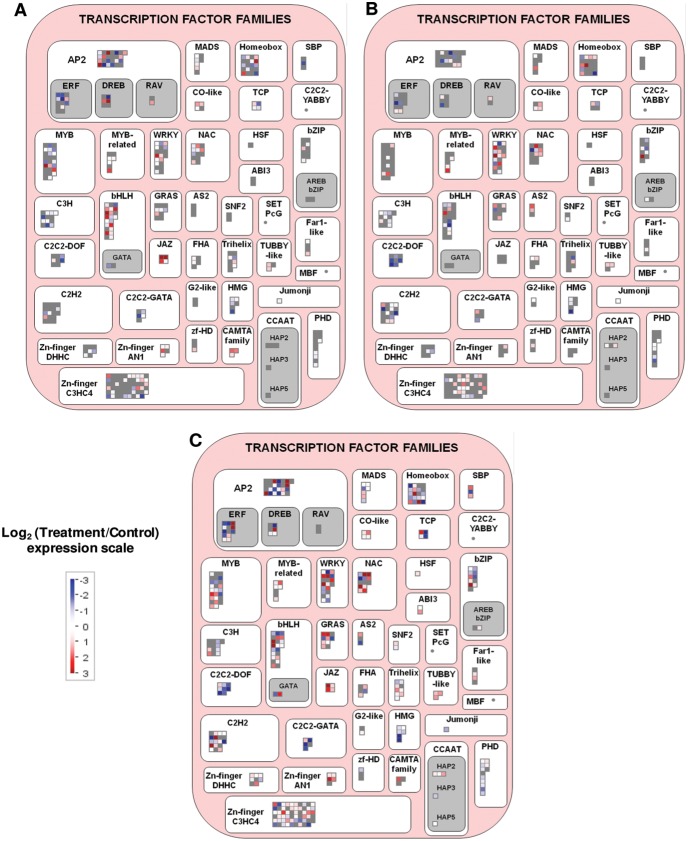
Mapman visualization of the significant genes in the ‘Transcription factor families’ functional category. A, MJ (methyl jasmonate); B, CD (cyclodextrins); C, CDMJ (cyclodextrins and methyl jasmonate). Resultant transcripts were considered after the average of significant redundant probesets expression values. Significant probesets according to a 5% FDR and *P*-value <0.05 for model variable in the corresponding control versus treatment 24 h series maSigPro comparison. Expression changes in the treatment normalized to these in the control are shown. Red, treatment up-regulated transcripts; blue, treatment down-regulated transcripts; grey, transcripts not significant in the treatment.

On the other hand, MJ induced both JAZ type and bHLH TFs, while CD significantly induced WRKY and NAC TFs, and repressed the C2C2-DOF (Figure 6 and Table S3). Moreover, CD and MJ applied together enhanced the induction of some WRKY, NAC, AP2 and JAZ type TFs (Figure 6). In relation to JAZ expression, the activation of jasmonic acid (JA) metabolism and signalling-related transcripts was higher in MJ-treated cells (Figure S5).

As regards hormonal regulation, all the treatments activated ethylene biosynthesis genes and repressed salicylic responsive genes (Figure S5). Finally, the CDMJ treatment also activated the expression of transcripts related with brassinosteroid signalling, and metabolism and transport of auxins (Figure S5).

## Discussion

### Cyclodextrins and/or methyl jasmonate induced the biosynthesis of defense-related proteins

The use of CD and MJ, separately or in combination as elicitors, has been proved to be very effective in cell reinforcing, stimulating the defensive arsenal in the apoplast-like extracellular medium of grapevine cell cultures through the accumulation of *trans-*R, and PR-proteins [2], [21], [24], [25]. In fact, functional categories enriched in clusters of transcripts from stilbenoid biosynthesis were found in CD and CD+MJ treatments (clusters 1 and 2, Figure 3). In addition, individual genes encoding PR-proteins were up-regulated in all treatments, more specifically two class IV chitinases (VIT_05s0094g00340 and VIT_05s0094g00330) and one *class I endochitinase* (VIT_03s0038g03400). In addition, one acidic *class III chitinases* (VIT_15s0046g01590) and one *thaumatin-like protein* (VIT_02s0025g04330) were also up-regulated in the MJ and CDMJ treatments (Table S3), and a protease inhibitor cystatin (VIT_00s0187g00040) in MJ-treated cells (Table S3). Moreover, defense responses related to oxidative stress were also induced by all treatments (Figure 3). These results are in accordance with those shown by Belchí-Navarro et al. [28] since the CD-mediated *trans-*R accumulation in *V. vinifera* cv Monastrell cell cultures was dependent on H_2_O_2_ production, and H_2_O_2_ levels increased significantly in the combined treatment with MJ after 24 h. In this sense, several peroxidases were down-regulated in CDMJ-treated cells (Table S3), what is consistent with higher H_2_O_2_ levels and higher *trans*-R accumulation in the combined treatment.

On the other hand, proteins that participate in the regulation of cellular redox that protect from the oxidative stress were also up-regulated in all treatments (cluster 6, Table S3). Amongst them, a *METHIONINE SULFOXIDE REDUCTASE* (VIT_19s0014g00170, Table S3) that reduces methionine sulfoxide in a thioredoxin-dependent reaction, provides a pathway to repair proteins damaged by reactive oxygen species in cells instead of having them be degraded [29].

### The up-regulation of genes involved in the biosynthesis of *trans-*resveratrol by cyclodextrin is enhanced in the presence of methyl jasmonate

Grapevine cell cultures elicited with CD and/or MJ displayed a significant inductive effect on biosynthetic processes related with phenylpropanoid and stilbenoid biosynthesis (clusters 1, 2, 11 and 6, Figure 3). Altogether, the biosynthesis of stilbenoids was up-regulated from the very early steps leading to the precursors, which involved genes coding for synthesis of phosphoenolpyruvate (*enolase*, VIT_17s0000g04540, Table S3) and D-erythrose 4-phosphate (functional category pentose-phosphate shunt, cluster 6, Figure 3), both responsible for feeding the shikimate pathway (Figure 5). In addition, the over-expression of genes encoding enzymes involved in the shikimate metabolism, aromatic amino acid, phenylpropanoid and stilbenoid biosynthesis was observed principally in the presence of CD either alone or in combination with MJ (clusters 1, 2, 11 and 6, Figure 3), thereby promoting the carbon flux toward these pathways (Figure 5) what is well-correlated with the high *trans*-R production found in these treatments (Figure 1). The simultaneous enrichment of functional categories corresponding to nitrogen metabolism, pantothenate and CoA biosynthesis and fatty acid metabolism within the transcripts up-regulated in all treatments (cluster 6, Figure 3) partly resulted from the presence in this cluster of genes encoding enzymes involved in the biosynthesis of acetyl-CoA and malonyl-CoA, such as ATP citrate lyase (VIT_05s0077g00950) and acetyl-CoA carboxylase (*ACC*, VIT_18s0001g04980), respectively (Table S3). Such elicitor response whose magnitude was greater in CD-treated cells and enhanced in CDMJ-treated cultures could be directed towards the production of phenylalanine and precursors of carbon skeletons for the biosynthesis of stilbenes and other phenylpropanoid compounds. Moreover, it is important to highlight a down-regulation of one gene encoding PEPC (VIT_01s0011g02740, Figure 5 and Table S3) in all treatments. Phosphoenolpyruvate is a key precursor in the production of oxaloacetate via PEPC, 4-coumaroyl CoA, via the shikimate-phenylpropanoid pathway, and also a precursor of malonyl-CoA, via pyruvate kinase, pyruvate dehydrogenase and ACC. Therefore, the down-regulation of PEPC might lead to a depletion of oxaloacetate in favor of phosphoenolpyruvate, which would provoke a critical change in the partition of carbon flux between primary and secondary metabolism since the excess of acetyl-CoA produced from pyruvate in CD- and CDMJ-treated cells would be used for biosynthesis of malonyl-CoA, which is extensively used for the production of stilbenes and flavonoids (Figure 5, [30]). Considering that up to a 20% of the carbon supplied to the cells as sucrose (20 g·l^−1^) is recovered as *trans*-R in the culture medium (around 4 g·l^−1^) after several days of elicitation [2], [31], the role of PEPC as a point to decide the fate of the incoming carbon could be of great relevance.

*PAL, C4H* and *4CL* genes encoding enzymes of the general phenylpropanoid biosynthetic pathway, and particularly these together with *STS* corresponding to stilbene biosynthesis were largely up-regulated by CD, and this response was intensified in the presence of MJ (Figure 4B and 4C and Table S3), and correlated with a high production of *trans*-R (Figure 1). Similar results have been found by Martínez-Esteso et al. [25] who observed that the addition of CD and CDMJ to *V. vinifera* cv Gamay cell cultures provoked a large increase in *trans*-R production which was correlated with an increase in STS protein abundance. Overall, the activation of both, phenylpropanoid precursors and stilbene gene expression by CD together with the enhancement of this activation in the presence of MJ, was highly correlated with the high levels of *trans*-R induced in grapevine cell cultures elicited with CD and MJ (Figure 1). Additionally, many transcripts coding for enzymes in other branches of the phenylpropanoid metabolism, including flavonoid and monolignol biosynthesis, were strongly up-regulated by the presence of CD and/or MJ (Table S3). These findings are in agreement with other studies performed in Arabidopsis [22] and grapevine [32]–[33] cell cultures.

On the other hand, the pathways for transport of *trans*-R to the extracellular medium in grapevine cells, being of utmost biotechnological relevance, are mostly unknown. In this sense, an increase in the transcript levels related to *MATE* and *ABC* transporters, which was more intense in cells treated with MJ alone or combined with CD than in CD-treated cells, was observed (Figure S4 and Table S3). In addition, some glutathione transferases, which are involved in the trafficking and accumulation of secondary metabolites such as anthocyanins [34]–[36] and in the sequestration of xenobiotics [37], were specifically up-regulated by MJ or CDMJ (cluster 11 in Figure 3 and Table S4). In agreement with our results, Martinez-Esteso et al. [25] observed that CD and MJ provoked an increase of glutathione transferases in *V. vinifera* cv Gamay cell cultures, with abundance profiles similar to those found for *STS* gene expression, and suggested the existence of a coordinated action between the biosynthesis and transport of *trans*-R in grapevine cells treated with CD and MJ. Future targeted studies are required to determine whether ABC or MATE transporters and glutathione transferases induced by CD and/or MJ (Table S3) would play a role in *trans*-R mobilization or sequestration.

### Regulatory cascades activated by cyclodextrins and/or methyl jasmonate

Consideration of the putative regulatory genes showing an altered expression in grapevine cell cultures elicited with CD and/or MJ, can help understanding the different responses that they trigger. The elicitation response triggered by MJ involves the JA signalling cascade, as it was shown in the functional analysis (Figure 3 and Table S4). Although jasmonate responses are controlled at the protein level, via ubiquitin-dependent proteolysis of JASMONATE ZIM-DOMAIN (JAZ) TF [38]–[39], MJ up-regulated *MYC2* and *MYC3* grapevine homologues (VIT_15s0046g00320 and VIT_02s0012g01320 in Table S2), which are the main factors triggering JA responses that directly interact with JAZ proteins in Arabidopsis [40]–[41]. Concurrently, three *JAZ* TF encoding genes (VIT_01s0146g00480, VIT_11s0016g00710 and VIT_17s0000g02230; Table S2), which are the main repressors of JA responses [38]–[39], were also up-regulated in response to MJ, suggesting a mechanism which is activated as a result of an increased sensitivity of the signalling pathway in response to the treatment. In fact, a positive self-regulating loop activating oxylipin and JA biosynthesis also seems to occur in the presence of MJ (Figure 3 and Table S4). This agrees with other studies showing that jasmonate biosynthesis and its signalling are interlinked by a positive feedback loop whereby jasmonates stimulate their own biosynthesis [42]. Besides, genes encoding other TFs like *WRKY* (VIT_11s0052g00450 and VIT_14s0108g01280), *NAC* (VIT_13s0019g05240), *ARF2* (VIT_17s0000g00320) and *MYB* (VIT_08s0007g07230) (Figure 6 and Table S3) were significantly activated only in the presence of MJ. Therefore, the cell responses transcriptionally activated by MJ may operate by inducing these TFs.

Bearing in mind that CD and CDMJ-mediated *trans*-R accumulation is held for several days, it is interesting to note that transcripts encoding proteins involved in putative early signalling events were up-regulated. For instance, the presence of CD up-regulated nine transcripts coding for protein receptor-like kinases and three transcripts for calmodulins, while the joint addition of CD and MJ up-regulated nineteen kinases and receptor-like kinases, and four calcium sensors (Table S3). Our studies on early signalling events using pharmacological approaches have pointed out the central role of phosphorylation/dephosphorylation cascades and calcium signalling in the production of *trans*-R in grapevine cell cultures [28]. Additionally, other TFs such as *MYB15* (VIT_05s0049g01020), three *NACs* (VIT_08s0007g07670, VIT_18s0001g02300 and VIT_19s0014g03290) and a *WRKY* (VIT_09s0018g00240) recently annotated as *VvWRKY30* [43], were up-regulated only in the presence of both elicitors (Figure 6 and Table S3), indicating that they could participate in the enhancement of the elicitation response when both compounds are added together. Indeed, *MYB15* and *MYB14* TFs specifically activate the promoter of *STS* genes in grapevine [15], indicating that the enhanced expression of *STS* genes in CDMJ-treated cells may result, at least in part, from the up-regulation of *MYB15*. Although the significant GrapeGen GeneChip VVTU9342_was previously annotated as *MYB14* (Table S2; [44]), the blast alignment of the probeset confirmed that it actually corresponds to the *MYB15* grapevine gene [45]. Unfortunately probesets representing the current *MYB14* gene (VIT_07s0005g03340) are missing from the GrapeGen GeneChip and therefore, its expression was not tested in our work. In the same way, *VvWRKY30* might be another *STS* expression-promoting TF in view that it was also co-induced with *STS* genes in grapevine leaves exposed to UV light, as well as in response to circadian rhythms that occur in grapevine fruits [46]–[47]. Furthermore, the identification of *MYB15*, *VvWRKY30* and *NAC* TFs and protein kinases that are specifically induced by the combined treatment suggests the synergistic activation of additional regulatory pathways due to crosstalk between the signalling cascades activated by CD and MJ. In this way, the induction of genes encoding protein kinases by CD might result in an enhancement of the elicitation responses activated by the two *WRKY* genes (cited above) induced only in the presence of MJ. Also, the gibberellin signalling repressor *VvGAI1* might be another node of crosstalk between CD and MJ signalling, resulting in increased jasmonate responses, since this *DELLA* gene (VIT_01s0011g05260) was specifically up-regulated in the presence of CD and MJ. In Arabidopsis, DELLA proteins bind to MYC2 preventing its interaction to JAZ repressors and allowing the activation of JA responses [48]–[49].

In parallel, a putative *MAPKKK* encoding gene (VIT_07s0031g00530) which was up-regulated in a similar extent in grapevine cell cultures treated with MJ alone or combined with CD, might participate in the enhancement of phosphorylation cascades triggered by CD.

The antagonisms and synergisms between JA and SA signals in the activation of pathogen responses are well documented [50]. These compounds coordinate the activation of a large set of defense responses and, when applied exogenously, they are able to induce resistance in both plants and cell cultures [11]. It is well known that most of the SA produced *in planta* is converted into SA glucoside by a pathogen-inducible SA glucosyltransferase [51]. This SA glucoside is actively transported from the cytosol to the vacuole in soybean and tobacco cells, where this inactive form of SA is stored [52]–[53]. Noticeably, we observed over-expression of genes encoding enzymes like UDP-glucose glucosyltransferase and UDP-glucose:SA glucosyltransferase, only in the presence of CD (Table S3). Moreover, SA-responsive transcripts were down-regulated upon elicitor treatment, and especially in the combined treatment (Table S3). These observations are consistent with the decrease in *trans*-R levels provoked by the addition of SA to grapevine cell cultures [21], suggesting that MJ and CD might increase *trans*-R biosynthesis through the attenuation of SA signalling induced by SA conjugation.

Finally, as ethylene biosynthesis is activated in all treatments, this molecule or its signalling may participate in the regulation of responses triggered by CD and/or MJ (Cluster 6, Figure 3). In fact, a great number of transcripts involved in ethylene signalling, like an *EIN4* putative ethylene receptor encoding gene (VIT_14s0081g00630), several ethylene response factors (*ERF,* VIT_07s0141g00690, VIT_10s0003g00580 and VIT_15s0021g01630; Table S3), and those from ethylene biosynthetic pathway were up-regulated in grapevine cells treated with CDMJ. In agreement with our results, Zamboni et al. [32] also observed the induction of a 1-aminocyclopropane-1-carboxylate oxidase in *V. riparia × V. berlandieri* cell cultures elicited with CD after 6 h of treatment.

### Growth-related gene expression is repressed upon elicitor treatment

Microarray analysis of grapevine cell cultures elicited with CD and/or MJ showed a specific down-regulation of genes involved in the control of cell wall modification (cluster 15, Figure 3 and Table S3). These results paralleled those of Pontin et al. [46], who showed a reduction in cell wall loosening-related gene expression concurrently to *STS* genes induction as part of the defense mechanism against UV-B in *V. vinifera* cv Malbec leaves. Our results also showed that genes encoding enzymes involved in microtubule-driven movement were down-regulated in all treatments (Table S3) although this effect was stronger in the presence of MJ (cluster 5, Figure 3 and Tables S3 and S4). It is well-documented that the plant cytoskeleton is a highly dynamic and versatile scaffold composed of microtubules and actin microfilaments that plays an important role in many aspects of cell growth, including cell division and expansion, and intracellular organization [54]–[55]. The down-regulation of genes coding for α- and β-tubulin, villin, and kinesin in cells treated with CD and/or MJ (Cluster 5, Figure 3 and Tables S3 and S4) might indicate an arrest in both cell division and expansion. In fact, Ebel et al. [56] observed that mRNA degradation of a β-tubulin in soybean cells elicited with *Phytophthora sojae* provoked a repression of cell growth, and a regulation of cell expansion controlled by the cytoskeleton has also been shown in grapevine cell cultures [57]. Also, a down-regulation of genes encoding enzymes involved in both microtubule-driven movement and vesicle-mediated trafficking (Table S3) was observed in MJ treatments. This result agrees with the functional role of cytoskeleton in cellular trafficking mechanisms, that regulates the movement of the vesicles and endosomes on actin filaments or microtubules [58]. Moreover, the presence of CD provoked a significant repression of genes related to DNA replication, translation and the regulation of the cell cycle, responses that were even more intensively repressed when grapevine cells were elicited with CDMJ. These results suggest that the presence of CD predominantly decreased cell growth and division processes. As regards to the regulation of the cell cycle, it is important to highlight that a cell division cycle protein 48 encoding gene (*CDC48*, VIT_10s0071g00680) was specifically down-regulated in the presence of CD (Table S3). In fact, CDC48 protein is an important regulator of the cell cycle, and is required for the cell-cycle commitment point via degradation of the G1-cyclin-dependent kinase inhibitor [59]. In the same way, CDC48 protein promotes cell proliferation in maize roots [60] and it is also down-regulated upon elicitation in other species like tobacco [61]. Taking into account these results, CD could reduce cell proliferation through the down-regulation of CDC48. In addition, a probable explanation of the strong repression of cell division triggered by CD and MJ might be the need of the cell to redirect all the available resources to the activation of a defense-related metabolism. CD and MJ also strongly down-regulated translation-related transcripts like a set of ribosomal proteins (Table S3) as it has also been reported in soybean and rice cell cultures treated with fungal pathogens [62]–[63]. Therefore, the arrest of the cell cycle and the decrease of the expression of a set of ribosomal proteins in the grapevine cells treated with both elicitors would lead to the suppression of protein translation, thus contributing to saving energy. Finally, the level of repression of these basic processes is well-correlated with the high levels of *trans*-R observed in the elicited grapevine cell cultures (Figure 1 and Table S4), indicated that CD and MJ activated the secondary metabolism in detriment of basic cell processes like primary metabolism and cell division.

## Experimental Procedures

### Plant material

*V. vinifera* cv Monastrell calli were established in 1990 as described by Calderón et al. [64]. Grapevine cell cultures derived from them have been routinely maintained by periodical subcultures as described by Belchí-Navarro et al. [21].

### Elicitor treatments

Elicitation experiments were performed in triplicate using 12 day old grapevine cell cultures. At this stage of cell development, 20 g of fresh weight of cells were transferred into 250 mL flask and suspended in 100 mL of fresh culture medium described by Belchí-Navarro et al. [21]. Then, cell cultures were maintained (110 rpm, 25°C) during 10 h at 25°C in darkness in a rotary shaker (110 rpm) and then, they were supplemented with 50 mM CD (Wacker Chemie, Spain) and/or 100 µM MJ (Duchefa, Spain). Control treatments without elicitors (C) were always run in parallel. All cell cultures were incubated up to 72 h under the same conditions described above. After elicitation, cells were separated from the culture medium by filtration, rapidly washed with cold distilled water, weighted and frozen at −80°C until use. The elicited culture medium was used for *trans*-R quantification.

### Quantification of *trans*-resveratrol in both culture medium and cells

For this, 20 µL of the spent medium were analyzed in a HPLC-DAD (Waters 600E, Waters 996) as described by Belchí-Navarro et al. (2012). In addition, 50 mg of freeze-dried cells were extracted overnight in 4 mL methanol at 4°C. The cell extract was diluted with water to a final concentration of 80% (v/v) methanol. Then, 20 µl of the diluted extracts was filtered (Anopore 0.2 µm) and analyzed in a HPLC-DAD (Waters 600E, Waters 996) as described by Bru et al. (2006) using a Spherisorb ODS2 C-18 column (250×4.6 mm, 5 µm). *trans*-R was identified at 304 nm and quantified by comparison with authentic standard of >99% purity (Sigma-Aldrich, Spain).

### Gene expression analyses

#### RNA isolation, cDNA synthesis and real-time quantitative PCR

To validate the microarray expression data were performed qRT-PCR experiments. For this purpose, Total RNA was isolated from frozen cells (0.5 g fresh weight) using the TRIZOL reagent (INVITROGEN, Spain) following the manufacture’s recommendations. The concentration of each RNA sample was measured using a NanoDrop ND-1000 spectrophotometer (NanoDrop Technologies). Only the RNA samples with a 260/280 ratio between 1.9 and 2.1 were used for the analysis. The integrity of RNA samples was also assessed by agarose gel electrophoresis. One µg of total RNA from each sample was reverse-transcribed by first-strand cDNA synthesis using MMLV RT (Invitrogen), according to the manufacturer’s instructions. SYBR Green PCR Core Reagents (Applied Biosystems) were used for qRT-PCR in an ABI PRISM 7500 instrument (Applied Biosystems). Gene specific primers (Table S1) were designed using the OligoAnalyzer 3.1 software (IDT, Integrated DNA Technologies) based on the corresponding probeset sequence from the custom GrapeGen GeneChip. For each primer pair, reaction efficiency estimates were derived from a standard curve generated from a serial dilution of pooled cDNA. For each gene, the expression levels were normalized with respect to grapevine EFα1 gene (Forward primer: GAACTGGGTGCTTGATAGGC; Reverse primer: AACCAAAATATCCGGAGTAAAAGA) used as reference control as described Lijavetzky et al. [27] and Reid et al. [65]. The same RNA samples used for the microarray hybridizations were used for the synthesis of the cDNA analyzed by qRT-PCR: three biological replicates of C, CD, MJ and the combined treatment (CDMJ) at 0 and 24 h. Correlation coefficients of 0 to 24 h expression ratios on each treatment normalized to the same expression temporal ratio in the control between microarray and qRT-PCR data were calculated for validation.

#### GeneChip hybridization

Samples of 0 and 24 h from each treatment were hybridized at the Genomics Unit of the National Biotechnology Centre (CNB-CSIC, Madrid). RNA integrity analysis was performed with an Agilents Bioanalyzer 2100. The custom GrapeGen Affymetrix GeneChip (A-AFFY-162 and GPL11004 ArrayExpress and GEO accession numbers, respectively), which contained 23,096 probesets, corresponding to 18,711 non-redundant grapevine transcripts [66] was processed as previously described [46].

#### Expression data preprocessing and Principal Component Analysis plot

The full GeneChip raw expression dataset is available on PlexDB [67] under the accession number VV44. Probeset signal values from all the microarray hybridizations were normalized together using Robust Microarray Average (RMA) [68] by RMA Express (http://rmaexpress.bmbolstad.com). A PCA [69] was directed over the full dataset after the average of redundant probesets expression values. Probeset redundancy was reduced according to the GrapeGen GeneChip 12Xv1 annotations version [44] using Babelomics preprocessing tools [70]. The three first Principal Components were analyzed using Acuity 4.0 (Axon Molecular Devices, http://www.moleculardevices.com).

#### Identification and clustering of differentially accumulated transcripts

Each treatment-time series was compared to the control in a maSigPro analysis conducted in Babelomics suite from RMA normalized expression data [70]–[71]. A 0.05 significance level after Benjamini and Hochberg multiple test adjustment and *P*-value <0.05 for model variable were applied. Besides, probesets were considered significant only when the expression difference normalized to the control was more than 2-fold in at least one treatment. RMA normalized data, maSigPro significance level and expression ratio for all probesets can be found in Table S2. The significant transcripts identified were grouped according to shared responses normalized to the control. SOM clustering [72] with Euclidean squared and Scale rows metrics was applied in Acuity 4.0 for the clustering. Fifteen groups were generated in a 5×3 clustering as estimated by gap statistics [73] in Acuity 4.0. Venn diagrams comparing up-regulated and down-regulated non-redundant transcripts among treatments were performed in Venny (http://bioinfogp.cnb.csic.es/tools/venny/index.html).

#### Functional analysis

Non-redundant GrapeGen 12Xv1 transcripts found within each expression profile cluster were analyzed in FatiGO [74] to search for significant enrichment of GrapeGen 12Xv1 functional categories [44]. Fisher’s exact test was carried out in FatiGO to compare each study list with the list of total non-redundant transcripts housed in the GrapeGen GeneChip. Significant enrichment was considered in case of *P*-value <0.05 after Benjamini and Hochberg correction for multiple testing. GrapeGen 12Xv1 MapMan pathways [44] were selected, taking into account significantly over-represented functional categories for gene expression responses to treatments, to be depicted in MapMan software [75]. An expression heatmap including differentially expressed genes in the biosynthetic pathway leading to stilbene production was performed in MeV version 4.8 [76].

## Supporting Information

Figure S1 **qRT-PCR expression validation of the microarray hybridization experiments.**
(DOCX)

Figure S2 **Treatments significantly regulated gene expression profiling summary.**
(DOCX)

Figure S3 **Mapman visualization of the significant genes in the ‘Amino acid metabolism’ pathway.**
(DOCX)

Figure S4 **Mapman visualization of the significant genes in the ‘Transport overview’ pathway.**
(DOCX)

Figure S5 **Mapman visualization of the significant genes in the ‘Hormone signalling’ pathway.**
(DOCX)

Table S1 **PCR primers used to amplify gene-specific regions for expression analyses.**
(XLS)

Table S2 **RMA normalized expression dataset. Log_2_ RMA GrapeGen GeneChip probesets signal in each sample.**
(XLSX)

Table S3 **Probesets significantly regulated 24 h after any treatment.**
(XLS)

Table S4 **Functional categories over-represented in each expression cluster of significantly regulated genes.**
(XLS)
