# Supplementary material for: Dissecting the Transcriptional Response to Elicitors in Vitis vinifera Cells
Source: PLoS One. 2014 Oct 14;9(10):e109777. doi: 10.1371/journal.pone.0109777 (PMC4196943; doi:10.1371/journal.pone.0109777)
Supplement: Figure S1 — qRT-PCR expression validation of the microarray hybridization experiments. (DOCX) [file pone.0109777.s001.docx]

**Figure S1. qRT-PCR expression validation of the microarray hybridization experiments.** MJ, methyl jasmonate; CD, cyclodextrins; CDMJ, cyclodextrins and methyl jasmonate. Comparison of gene expression values of different genes reported by the GrapeGen *V. vinifera* Affymetrix GeneChip® and by quantitative real-time RT-PCR (qRT-PCR). Log_2_ [(treatment at 24 h/treatment at 0 h)/ (control at 24 h/control at 0 h)] expression ratios calculated from microarray data (y-axis) are represented against the same ratio obtained from qRT-PCR data (x-axis). Linear regression analyses (R^2^ values) are also shown.
