# Supplementary material for: Dissecting the Transcriptional Response to Elicitors in Vitis vinifera Cells
Source: PLoS One. 2014 Oct 14;9(10):e109777. doi: 10.1371/journal.pone.0109777 (PMC4196943; doi:10.1371/journal.pone.0109777)
Supplement: Figure S2 — Treatments significantly regulated gene expression profiling summary. (DOCX) [file pone.0109777.s002.docx]

**Figure S2**. **Treatments significantly regulated genes expression profiling summary.** C, control; MJ, methyl jasmonate; CD, cyclodextrins; CDMJ, cyclodextrins and methyl jasmonate. Clustering of significantly regulated probesets (5% FDR and *p*-value<0.05 for model variable in the corresponding maSigPro control versus treatment time series comparison, and more than 2-fold change in at least one treatment normalized to the control). 15 clusters were generated in a 5X3 SOM clustering as an optimal number of groups assessed by a GAP analysis. Magenta and green intensity, level of up-regulated or down-regulated expression respectively, 24 h after the treatment and normalized to the control; Black, transcripts not significantly regulated by the treatment. The numbers in brackets indicate non-redundant transcripts / total probesets included in the cluster. Number at the left upper side, cluster profile: 1, CDMJ specifically up-regulated transcripts; 2, CD and CDMJ up-regulated transcripts; 3, MJ up-regulated transcripts; 4, MJ down-regulated transcripts; 5, MJ and CDMJ down-regulated transcripts; 6, Three treatments up-regulated transcripts; 7, Transcripts more highly up-regulated in MJ and CD single treatments than in CDMJ; 8, MJ up-regulated and other treatments down-regulated transcripts; 9, CD specifically down-regulated transcripts with CDMJ intensified repression; 10, CDMJ specifically down-regulated; 11, MJ and CDMJ up-regulated transcripts; 12, MJ specifically up-regulated transcripts; 13, CD specifically down-regulated transcripts; 14, CD and CDMJ down-regulated transcripts; 15, Three treatments down-regulated transcripts.
