# Supplementary material for: Dissecting the Transcriptional Response to Elicitors in Vitis vinifera Cells
Source: PLoS One. 2014 Oct 14;9(10):e109777. doi: 10.1371/journal.pone.0109777 (PMC4196943; doi:10.1371/journal.pone.0109777)
Supplement: Figure S5 — Mapman visualization of the significant genes in the ‘Hormone signalling’ pathway. (DOCX) [file pone.0109777.s005.docx]

**Figure S5**. **Mapman visualization of the significant genes in the ‘Hormone signalling´ pathway.** **A**, MJ (methyl jasmonate); **B**, CD (cyclodextrins); **C**, CDMJ (cyclodextrins and methyl jasmonate). Resultant transcripts were considered after the average of significant probesets redundancy. Significant probesets according to a 5% FDR and *p*-value<0.05 for model variable in the corresponding control versus treatment 24 h series maSigPro comparison. Expression changes in the treatment normalized to these in the control. Red, treatment up-regulated transcripts; blue, treatment down-regulated transcripts; grey, transcripts not significant in the treatment.
